# MicroRNA: a new and promising potential biomarker for diagnosis and prognosis of ovarian cancer

**DOI:** 10.7497/j.issn.2095-3941.2015.0024

**Published:** 2015-12

**Authors:** Manish K. Pal, Shyam P. Jaiswar, Vinaya N. Dwivedi, Amit K. Tripathi, Ashish Dwivedi, Pushplata Sankhwar

**Affiliations:** ^1^Department of Obstetrics and Gynecology, King George Medical University, Lucknow, UP 226003, India; ^2^Biochemistry and Molecular Biology Laboratory Center for Advanced Study in Zoology, Department of Zoology, Banaras Hindu University, Varanasi, UP 221005, India; ^3^Endocrinology Division, Central Drug Research Institute, Lucknow, UP 226001, India; ^4^Photobiology Division, Indian Institute of Toxicology Research, MG Marg, Lucknow, UP 226001, India

**Keywords:** MicroRNAs (miRNA), biomarker, chemoresistance, detection, RT-PCR

## Abstract

Epithelial ovarian cancer (EOC) is the leading cause of death among all gynecological malignancies. Despite the technological and medical advances over the past four decades, such as the development of several biological markers (mRNA and proteins biomarkers), the mortality rate of ovarian cancer remains a challenge because of its late diagnosis, which is specifically attributed to low specificities and sensitivities. Under this compulsive scenario, recent advances in expression biology have shifted in identifying and developing specific and sensitive biomarkers, such as microRNAs (miRNAs) for cancer diagnosis and prognosis. MiRNAs are a novel class of small non-coding RNAs that deregulate gene expression at the posttranscriptional level, either by translational repression or by mRNA degradation. These mechanisms may be involved in a complex cascade of cellular events associated with the pathophysiology of many types of cancer. MiRNAs are easily detectable in tissue and blood samples of cancer patients. Therefore, miRNAs hold good promise as potential biomarkers in ovarian cancer. In this review, we attempted to provide a comprehensive profile of key miRNAs involved in ovarian carcinoma to establish miRNAs as more reliable non-invasive clinical biomarkers for early detection of ovarian cancer compared with protein and DNA biomarkers.

## Introduction

Epithelial ovarian cancer (EOC) is the leading cause of death in women among all gynecological malignancies, accounting for about 5% of all cancers and 4.2% of all cancer deaths in women worldwide^1^^,^^2^. Recent prevalence data suggested that it is the most common type of ovarian cancer. EOC is characterized as a heterogeneous group of disease mainly divided into four different histologic subtypes, such as serous, mucinous, clear cell, and endometrioid carcinoma^3^^,^^4^. These complexities are further enhanced by the fact that each subtype has distinct molecular and genetic profiles and behaves and responds differently to treatment, thereby imposing a great hurdle for designing a common therapeutic regimen to treat this devastating illness.

Despite the advances in detection and cytotoxic therapies, only a modest increase in the survival rate beyond five years after initial diagnosis of ovarian cancer has been achieved. The high mortality of patients suffering from this disease is attributed to various factors, including the lack of any specific symptoms in early stages of ovarian cancer; late diagnosis, which poses difficulty in designing an intervention; and the development of chemoresistance in cancer cells. Therefore, improved screening strategies for EOC detection at early stage, as well as effective treatment for advanced stages of ovarian cancer patients, are necessary. In addition, novel biomarkers, particularly a distinct and specific biomarker for each subtype of ovarian cancer that can help in detection at the earliest stage, should be searched and developed, which will serve for designing better and more concentrated treatment strategy for ovarian cancer. Unfortunately, despite comprehensive and multifaceted scientific efforts worldwide, development of biomarkers with ideal specificities and absolute sensitivities remains a challenge. During the last few decades, several attempts to develop serum-based biomarkers for early detection of ovarian cancer have gained limited success because of their low sensitivities and specificities. Therefore, identifying and developing an alternative, non-invasive, sensitive, and more specific biomarker to detect ovarian cancer at the initial stages is necessary for the improved management of ovarian cancer patients. Fortunately, despite the limitations, recently discovered small RNAs, termed microRNAs (miRNAs), have the potential to serve as ideal non-invasive biomarkers for ovarian cancer.

## Current status of clinical applications of some important biomarkers for ovarian cancer

The poor diagnosis of ovarian cancer is mainly attributed to the lack of symptoms in the early stages of the disease, which can lead to the occurrence of distant metastasis. Incessant technological advancements in genomics and proteomics have identified multiple molecular biomarkers for ovarian cancer diagnosis. These biomarkers are broadly categorized into gene-based, protein-based, and miRNA-based biomarkers. Gene-based biomarkers measure inherited mutations, epigenetic changes (primarily DNA methylation and histone modifications), and gene expression levels of multiple genes involved in gene regulation. Hereditary germline mutations in genes, such as breast cancer 1/2 (*BRCA1* and *BRCA2*) and DNA mismatch repair genes, primarily *hMLH1* and *hMSH2*, have been detected in at least 10% of all EOCs. Therefore, genetic testing for mutations of these genes may help in potentially identifying patients with higher risk of developing ovarian cancer and designing intervention strategies to reduce the risk of ovarian cancer. Given the development of microarray technology, gene expression profiling enables the rapid comparison of gene expression between normal and malignant cells and identification of genes that are differentially regulated during cancer development.

Carbohydrate antigen 125 (CA-125) is the most widely used protein-based clinical biomarker for the diagnosis of ovarian cancer. Elevated CA-125 serum levels have been detected in about 80%-85% of women with advanced ovarian cancer, but only 50% of patients with stage I ovarian cancer will have an elevated CA-125 level, suggesting that it is neither sufficiently sensitive nor specific for early detection for ovarian cancer. Therefore, CA-125 is mostly used to follow up the stage-wise progression of ovarian cancer in patients with established ovarian cancer^5^; other important protein biomarkers for ovarian cancers include leptin, prolactin, osteopontin, and insulin-like growth factor II (IGF-II)^6^.

Another important protein biomarker for early detection of ovarian cancer is human epididymis protein 4 (HE4), a secreted glycoprotein product of the *WFDC2* gene. Elevated levels of HE4 have been reported in over 50% of ovarian cancer patients whose tumors do not express CA-125, indicating that HE4 has better potential in monitoring ovarian cancer compared with CA-125. HE4 also has better capacity to distinguish benign and malignant tumors, so it is more useful in identifying early stages of diseases than CA-125, which is also not cancer-specific and can even be elevated in various benign conditions. Many ovarian cancer screening research studies have established that combinatorial biomarker strategy (i.e., multiplexed biomarker approach implying a subset of markers) is more reliable, sensitive, and specific than using a single protein biomarker^7^. For example, a recent study identified the 5-marker panel for early detection of ovarian cancer that includes five serum biomarkers, namely, macrophage-stimulating protein alpha, tissue inhibitor of metalloproteinases-4, platelet-derived growth factor receptor alpha (PDGF-R alpha), osteoprotegerin, and CA-125. This panel showed an AUC of 0.98 compared with that of CA-125 alone (0.87) and correctly identified 100% ovarian cancer and 95% healthy control samples, providing the total correct agreement of 96.6%^8^.

A particular disadvantage associated with protein biomarkers is their limited diagnostic value because of their low sensitivity and specificity, high cost, and inconvenience. Therefore, novel and more specific biomarkers with greater diagnostic value should be identified and developed, particularly for the early detection of EOC to minimize the mortality rate and improve survival in ovarian cancer patients. Fortunately, recent evidence indicated that miRNA biomarkers can cope with the flaws of protein biomarkers and serve as more reliable biomarkers for EOC.

## MiRNAs: a novel and more reliable biomarker for early detection of ovarian cancer

MiRNAs are a novel class of small (18-24 nucleotides long), evolutionarily conserved non-coding RNA molecules that control various physiological and pathological functions by regulating gene expression at the post-transcriptional level^9^^,^^10^. miRNAs were originally discovered in *Caenorhabditis elegans*, and they are found in genomes of most eukaryotes, including humans^11^^-^^13^. Recent studies have suggested that miRNAs account for about 1%-5% of the human genome and regulate at least 30% of protein-coding genes^14^^-^^18^. To date, about 940 distinct miRNA molecules have been identified within the human genome^19^^-^^22^.

To improve the survival rate in ovarian cancer patients, highly specific and more sensitive biomarkers should be discovered and developed for ovarian cancer screening and early detection, as well as for survival prediction by assisting monitoring of response to chemotherapeutic drugs. For this purpose, miRNAs as biomarkers offer great tools to evaluate cancer development. They play essential roles in nearly all cellular pathways governing human malignancies, such as carcinogenesis, cancer progression, cell invasion and metastasis, cell survival, and response to therapeutic drugs. In addition, miRNAs also act as oncogenes and tumor-suppressor genes; thus, they have a large potential to serve as promising biomarkers for EOC^23^^,^^24^. Furthermore, miRNA biomarkers are more sensitive and specific than any other biomarkers examined for the diagnosis and prognosis of EOC.

### Genomic units of miRNAs and their roles in ovarian cancer: special focus on miR-200 and let-7 families

**Figure 1:**
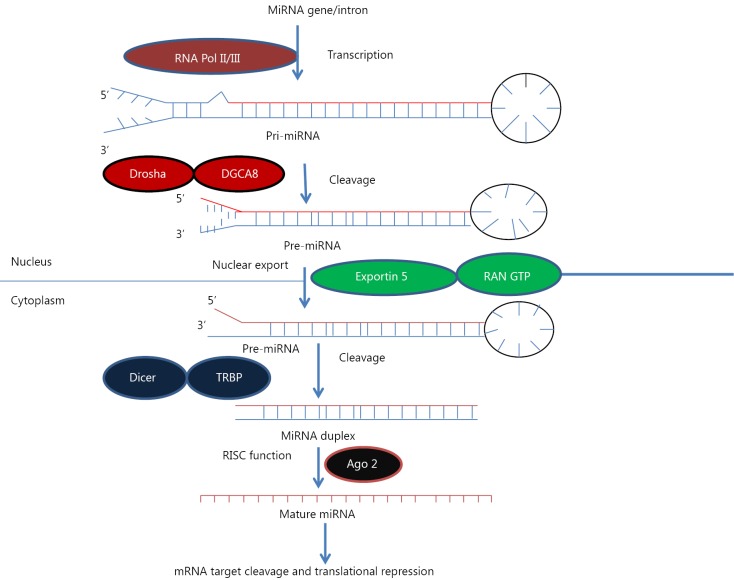
MicroRNA biogenesis. MiRNA genes are transcribed by RNA polymerase II or III to generate pre-miRNA and cleavage of the pre-miRNA by the microprocessor complex Drosha-DGCR8 (Pasha) in the nucleus to form precursor miRNA. Precursor miRNA is exported from the nucleus to cytoplasm by Exportin-5-Ran-GTP. In the cytoplasm, the RNase Dicer, in complex with the double-stranded RNA-binding protein TRBP, cleaves the pre-miRNA hairpin to its mature length. The miRNA-RISC complex then associates with target mRNA, resulting in repression of the target gene by promoting mRNA degradation and/or translational repression.

All miRNA genes have well-defined transcriptional units in the genomes. In mammals, miRNAs may be found at multiple locations, such as within introns and exons of protein coding genes, intergenic regions, noncoding genes, repetitive regions, and transposable elements, and they are transcribed similar to mRNAs^25^. miRNAs located in intergenic regions or in other annotated genes are in antisense orientation, whereas intragenic miRNAs can be oriented in both sense and antisense directions^26^. Interestingly, about 50% of annotated human miRNAs are associated with cancer and located at fragile sites, which are areas of the genome associated with cancer (**Figure 1**)^27^^,^^28^.

Given that this article does not cover the genomic units of all miRNAs and their alterations in ovarian cancer, most of our discussion will largely be confined to the miR-200 and let-7 family of miRs. Thus, only the genomic units of these two miRs are described. The miR-200 family of miRNAs, which are abundantly expressed in epithelial tissues, comprises five members (miR-200a, miR-200b, miR-200c, miR-141, and miR-429). These miRNAs are arranged in two clusters along the human genome. MiR-200a, miR-200b, and miR-429 are clustered on chromosome 1, whereas miR-200c and miR-141 are located on chromosome 12^29^. Several studies have reported changes in the expression of various members of the miR-200 family and suggested their possible roles in the pathogenesis of ovarian cancer. The miR-200 family is among the most significantly overexpressed miRNAs in EOC. Up-regulated expression of miR-200a and miR-200c has been reported in three types of ovarian cancers: serous, endometrioid, and clear cell carcinomas. However, miR-200b and miR-141 are up-regulated only in endometrioid and serous subtypes, suggesting that the role of the miR-200 family in ovarian carcinoma is more complicated and diverse than initially thought^30^.

The let-7 (lethal-7) family of miRNA, consisting of 13 different miRNAs, is located and distributed on nine different chromosomes in humans^31^^,^^32^. The expression of the let-7 family is significantly reduced in multiple human cancers. Low expression level of let-7 is associated with poor survival of cancer patients^33^^,^^34^. The let-7 family of miRNAs suppresses multiple ovarian cancer oncogenes, such as KRAS, HRAS, c-MYC^35^, and HMGA-2 ^36^. Notably, the genomic region harboring let-7a-3/let-7b locus was deleted in 44% of ovarian cancer samples studied. However, restored expression of let-7b significantly reduced ovarian tumor growth both *in vitro* and *in vivo*.

## MiRNAs are critical regulators of ovarian cancer from pathogenesis to cancer cell survival

MiRNAs perform diverse functions by modulating a broad range of gene expression patterns during development and tissue homeostasis, as well as in the pathogenesis of disease conditions. In ovarian cancer, miRNAs are involved in various cellular functions ranging from carcinogenesis, cell cycle, apoptosis, proliferation, invasion, and metastasis to development of chemoresistance. The role of miRNAs in ovarian cancer has been explored mainly through expression profiling of miRNAs in different cancer types. Various recent studies have reported global and individual miRNA expression patterns in different types of cancers. The first study linking miRNAs and cancer was reported in chronic myelogenous leukemia, in which miR-15 and miR-16 are deleted or down-regulated in tumor patients^37^. After this pioneer study, various research groups across the globe started to evaluate the role of miRNAs and their deregulations in various cancers. The deregulation of cancer-related miRNAs is due to several factors, including chromosomal rearrangements, alterations in genomic copy numbers, epigenetic modifications, abnormal maturation pathways and their regulation by transcription factors, and miRNA-miRNA interactions^38^. miRNA deregulation has been reported in almost all type of cancers, including ovarian cancer. Various miRNA species are differentially expressed among different histotypes in ovarian cancer^39^. These aberrantly expressed miRNAs promote tumor development by inactivating tumor suppressor genes and/or activating oncogenes, a common feature of all ovarian cancers^40^^,^^41^.

## Molecular signaling pathways involved in miRNA-mediated regulation of EOC

**Figure 2:**
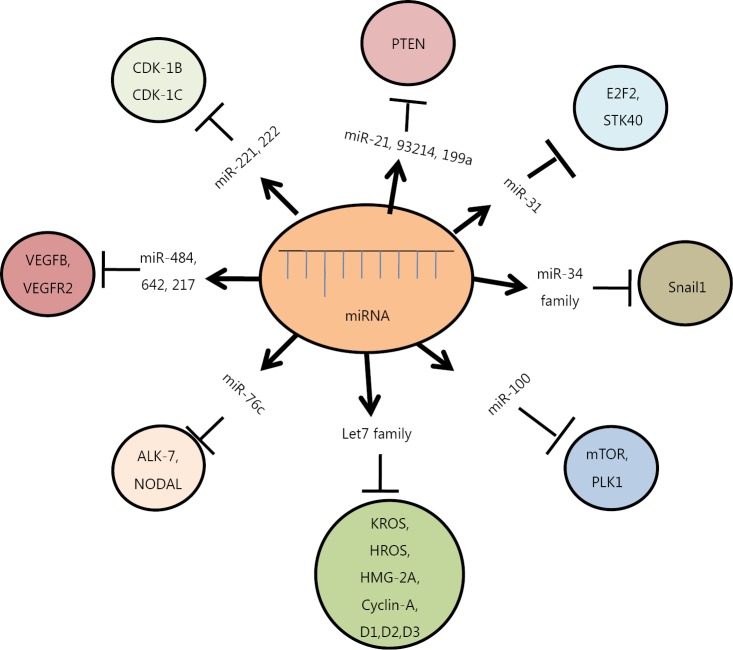
Diagrammatic representation of different miRNAs and their target genes. These miRNAs modulate various cellular pathways, either by upregulation or down-regulation of their respective target genes, ultimately causing cancer.

MiRNAs regulate diverse molecular signaling pathways of ovarian cancer pathogenesis by interacting with multiple target genes, and they modulate the functions of these genes across diverse cancer types. The transforming growth factor-β (TGF-β) signaling pathway is one of the best characterized pathways known to play a crucial role in ovarian cancer progression, particularly in epithelial-mesenchymal transition (EMT), through modulation by miR-181a. The key target genes of the TGF-β signaling pathway are the two receptor-regulated SMADs, namely, Smad2 and Smad3, including Smad7. TGF-β also plays a key role in EMT by repressing E-cadherin expression in epithelial cancer cells (the TGF-β/SMAD signaling pathway). Other critical pathways involved in miRNA regulation of EOC include the PI3K/AKT pathway, G-PCR signaling pathway, Wnt/β-catenin pathway, and ERK5 pathway^42^. Let-7 targets several oncogenes, such as c-Myc, ras, high-mobility group A (HMGA), Janus protein tyrosine kinase (JAK), signal transducer and activator of transcription 3 (STAT3), and NIRF. Another study has suggested that let-7a plays critical roles in tumorigenesis, proliferation, and invasion, possibly by regulating the cell cycle through the NIRF/p53/p21/CDK signaling pathway^43^. EOC is also characterized by alterations in epidermal growth factor receptor (EGFR), PI3K/AKT/mTOR signaling, and mutations or epigenetic losses of BRCA1/2, PTEN, and TP53^44^. Aside from the IL-6R-JAK-STAT3 pathway, nuclear factor kappa-B pathway, VEGF pathway, and other pathways have also been reported to regulate various aspects of ovarian carcinoma^45^ (**Figure 2**).

## MiRNAs control EMT

MiRNAs are involved in ovarian cancer initiation and progression^46^. Aberrant expression of miRNAs plays a critical role in cancer etiology and affects cellular functions by modulating the functional genome. Thus, identification of their target genes has gained significant interest for better management of ovarian cancer.

In the pathogenesis of ovarian cancer, transformation of epithelial cells to mesenchymal cells marks the inception of cancer development and invasion. This transformation (EMT) is characterized by a series of reversible events involving molecular reprogramming and phenotypic changes in cells, leading to dedifferentiation of polarized, immotile epithelial cells to motile mesenchymal cells; this dedifferentiation occurs when the level of E-cadherin protein, a class of type-1 transmembrane protein responsible for maintaining identity of epithelial cells, is reduced^47^. The miR-200 family of miRNAs plays an important role in this transition by targeting ZEB-1 and ZEB-2, the transcriptional repressor of E-cadherin genes^48^. MiR-200 family members include miRNA-200a, miRNA-200b, miRNA-200c, miR-141, and miR-429. Different members of the miR-200 family influence EMT transition and cancer progression in different manners by affecting multiple target genes and regulatory pathways involved in ovarian carcinoma. For example, a strong and positive correlation between the expression of E-cadherin and miR-200c has been reported in ovarian cancer tissues. Furthermore, the miR-200 family members have also been found to suppress ZEB1 and ZEB2, resulting in subsequent suppression of EMT^49^. Concomitantly, overexpression of miR-200 a/b/c and/or miR-141 down-regulates ZEB1/2 levels, leading to higher levels of E-cadherin and epithelial phenotype. By contrast, another study reported that ZEB1/2 can also inhibit the expression of E-cadherin by blocking the transcription of miR-200 family members by binding to clusters of the miR-200 promoter. For EMT induction, the cells are activated by external stimuli, such as TGF-β or PDGF-D, which trigger the expression of transcriptional repressor ZEB1/2 and decrease the expression of miR-200^50^. Nonetheless, miR-200 family members also control the expression of β-tubulin III, as well as its response with paclitaxel-based therapy and progression-free survival in ovarian cancer patients. The down-regulation of the miR-200 family causes increased expression of β-tubulin III, which leads to the development of chemoresistance in ovarian cancer patients^51^. Reduced expression of miR-200c is associated with recurrence of ovarian cancer. In addition, miR200 overexpression also significantly inhibits ovarian cancer cell invasiveness and metastasis by down-regulating MMP3, possibly through ZEB1/pSMAD3^52^.

MiR-34-a/b/c induced by p53 is down-regulated in ovarian cancer. The p53 mutation or loss-of-function promotes EMT of cancer cells by increasing the expression of Snail1 protein as the miR-34 family of miRNAs suppresses Snail1 activity when it binds to highly conserved 3’-untranslated region in Snail1 and its regulatory molecules, including β-catenin, LEF1, and Axin2. Therefore, mutated p53 down-regulates miR-34a/b/c to maintain the level of Snail1 protein, which is a zinc-finger transcriptional repressor that regulates EMT programs of cancer cells^53^^,^^54^. The possible molecular mechanisms underlying their down-regulation include aberrant promoter methylation of miRNA genes or any alterations in their copy number^53^.

Oxidative stress produced during the progression of ovarian cancer also affects various aspects of cancer proliferation and therapeutic approach by regulating the expression of miRNAs. MiR-141 and miR-200a modulate the oxidative stress response by targeting p38α. On the one hand, overexpression of these two miRNAs mimics p38α deficiency and increases tumor growth; on the other hand, it improves the response to chemotherapeutic agents in mouse models. High-grade human ovarian adenocarcinomas with increased accumulation of miR-200a show decreased levels of p38α and associated oxidative stress^55^. Given that the miR200a-dependent stress signature has fair correlation with improved survival of patients in response to treatment, it certainly can serve as a potential biomarker to predict clinical outcome in ovarian cancers. In addition, apart from promoting tumor growth, oxidative stress also sensitizes tumors for treatment and significantly limits the success of antioxidants in clinical trials^55^.

## MiRNAs critically regulate tumor growth, angiogenesis, and cancer cell survival in ovarian cancer

Down-regulation of certain miRNAs promotes tumor growth and survival of cancer cells by inhibiting apoptosis, inducing metastasis/angiogenesis, and developing chemoresistance against many drugs in ovarian carcinoma. Reduced levels of these miRNAs may be insufficient to check the upregulation of several oncogenes, thereby enhancing tumor development. Among the various down-regulated miRNAs, the let-7 family of miRNAs, which targets embryonic genes, such as *HMAG2*, *Mlin-41*, and *IMP-1*, is universally and significantly down-regulated in all expression profiles of ovarian cancer. The early phase of tumorigenesis resembles embryonic development, which involves re-expression of embryonic mesenchymal genes and de-differentiation of cancer cells, and it is caused by down-regulation of the let-7 family of miRNAs^36^. Let-7 suppresses several oncogenes, such as KRAS, HRAS, and c-MYC, in ovarian cancer^35^. The reduced level of let-7 in ovarian cancer cells causes upregulation of these oncogenes by enhancing tumor growth and cell survival^56^, which ultimately leads to poor survival of cancer patients^57^.

MiR-214 is responsible for the constitutive activation of the PTEN/ AKT pathway, leading to chemoresistance in different types of tumors, including ovarian cancer^58^. The increased expression of miR-214 is involved in cisplatin resistance in ovarian cancer by down-regulation of PTEN protein and activation of the PI3K/AKT/mTOR pathway, thereby enhancing cell survival^58^. In addition, miR-21 located on chromosome 17q23 is overexpressed in most cancers and plays a critical role in neoplastic transformation, invasion, and metastatic processes. MiR-21 inhibits apoptosis in cancer cells by targeting PTEN and PDCD4 and activating the AKT pathway^59^. Similarly, down-regulation of miR-100, a tumor suppressor miRNA, mediates increased sensitivity to everolimus in most ovarian cancer cell lines and tissues primarily through the repression of mTOR-AKT signaling^60^^,^^61^. Ye *et al.*^62^ showed that miR-376c promotes cell proliferation, survival, and spheroid formation by suppressing activin receptor-like kinase 7 (ALK7) and its ligand Nodal, which both induce apoptosis in human EOC cells. Chemosensitive and chemoresistant ovarian tumors have shown differential expression of ALK7 and miR-376c. Overexpression of miR-376c blocks cisplatin-induced cell death and enhances cell survival. Another study showed that miRNA-93 stimulates tumor growth and angiogenesis by targeting the integrin-β8 gene^63^ (**Figure 2**).

Through a gain-of-function screen using the miRNA mimic library (319 miRNA species) to identify various miRNA species affecting cell proliferation in human EOC cells, A2780 has shown the overexpression of miR-124a, miR-192, miR-193a, and miR-193b and subsequent inhibition of BrdU incorporation in A2780 cells, indicating that these miRNAs affect the cell cycle. Overexpression of miR-193a and miR-193b induces the activation of caspase 3/7, which leads to apoptotic cell death in A2780 cells. MiR-193a also potentially targets the *ARHGAP19*, *CCND1*, *ERBB4*, *KRAS*, and *MCL1* genes and decreases the level of MCL1 protein by binding to 3’-UTR of its mRNA^64^. MiRNAs also play intricate roles in the development of drug resistance in EOC. Most notably, hsa-miR-152, hsa-miR-200a-3p, hsa-miR-381, and hsa-miR-429 are differentially expressed between drug-sensitive and drug-resistant tissues^65^. Integrated genomic analyses have predicted the crucial involvement of eight key miRNAs, including miR-506, miR-141, and miR-200a, in regulating 89% of the targets in the network controlling survival of cancer cells in serous ovarian cancer^66^. Concomitantly, these findings directly implicated the intricate involvement of miRNAs in gene regulation related to tumor growth and cell survival, suggesting that miRNAs could potentially be used for therapeutic purposes in ovarian cancer.

## MiRNAs regulate cell cycle in ovarian carcinoma

MiRNAs also include various cell cycle genes as their targets, and modulating their expression plays significant roles in cell cycle progression in various histotypes of ovarian cancers. The let-7 family of miRNAs regulates several cell cycle genes, such as *Cyclin-A*, *D1*, *D2*, *D3*, and *HMG-2A*^56^. MiRNA-31 observed in serous ovarian cancer cell lines and tissues inhibits the expression of multiple cell cycle regulators, such as E2F2 and STK40, which are repressors of p53-mediated transcription^67^. The enhanced expression of miR-221 and miR-222, which are located on X-chromosome, inhibits apoptosis by down-regulating cell cycle genes, namely, CDK1B (p27) and CDK1C (p57)^68^. Furthermore, miR-210 is the most prominent miRNA that is consistently stimulated under hypoxic conditions, and it is regulated by the HIF signaling pathway. MiR-210 also regulates the e2f transcription factor 3 (e2f3), a key protein in the cell cycle, as shown by both biocomputational analysis and *in vitro* studies. MiR-210 down-regulates the expression of E2F in ovarian cancer patients, indicating that miR-210 plays a crucial role in tumor onset as a key regulator of the hypoxic response of tumor cells and provides a link between hypoxia and cell cycle regulation^25^. More recently, the dysregulation of five miRNAs has been linked to the significant enrichment of the cell cycle, revealing that the MAPK signaling pathway is highly involved in ovarian carcinogenesis; these findings suggested that these miRNAs may function as diagnostic and prognostic  biomarkers^69^ (**Figure 2**).

## MiRNAs as a powerful diagnostic tool for ovarian cancer

Among the various curative and preventive measures considered, early detection has long been the key to successful treatment of multiple life-threatening diseases, including ovarian cancer. Early detection underlies the foundation for better management and intervention of ovarian cancer at its primary stage before it spreads and affects other healthy tissues of the body. The aberrant expression pattern of miRNAs can be a powerful tool to diagnose ovarian cancer in its earliest stages. Strengthening this view, Iorio *et al.*^30^ reported the aberrant expression of miRNA in ovarian cancer and found that miR-141 and miR-200a/b/c are the most significantly overexpressed, whereas miR-199, miR140, miR-145, and miR-125b are significantly down-regulated. MiR-200a and miR-200c are overexpressed in all histologic groups, such as serous, endometrioid, clear cell, and mixed carcinoma, whereas miR-200b and miR-141 are endometrioid and serous-specific. A very recent study has also reported significant down-regulated expression of serum miR-145 in patients with malignant ovarian cancer and benign ovarian cancer compared with that in healthy controls, and results suggested that miR-145 can potentially serve as an outstanding biomarker for the detection of ovarian and other human cancers^70^.

Hypomethylation of miRNA genes is the key epigenetic mechanism for upregulation of miR-21, miR-203, and miR-205 compared with normal ovary^30^. Another study has reported that upregulation of miRNAs is due to the amplification of miRNA genes in ovarian cancer^41^. Among the 35 miRNAs analyzed, four miRNAs (miR26, miR182, miR103, and miR26a) were significantly up-regulated, whereas two miRNAs (let-7d and miR-127) were highly down-regulated. These studies pointed out that alterations in copy number and epigenetic silencing of miRNA genes are the two mechanisms of aberrant expression of miRNAs in ovarian cancer^41^. Furthermore, miR-30c, miR-30d, and miR-30e are frequently up-regulated, whereas miR-493 is down-regulated in ovarian carcinomas compared with that in normal HOSE cell lines. However, miR-30a has been identified as a clear cell-specific miRNA in ovarian cancer^71^. In a recent study, comparative and detailed miRNA expression profiling was performed in various tissues and malignant ovarian tumors using quantitative real-time PCR (qPCR) to explore the relationship between frequently deregulated miRNAs and clinicopathologic variables, response to therapy, survival, and their association with Her-2/neu status in ovarian carcinomas. This study clearly established that expression levels of the four miRNAs (miR-30c, miR-30d, miR-30e-3p, and miR-370) is significantly higher in ovarian carcinoma than that in benign ovarian tissue, whereas, three miRNAs (miR-181d, miR-30a-3p, miR-532-5p) are significantly different between borderline and ovarian carcinoma. Most importantly, miR-370, which is highly up-regulated in early stage of ovarian cancer, can be used as a biomarker for early detection of ovarian cancer. In addition, the down-regulation of miR-30c, miR-30d, miR-30e-3p, and miR-532-5p is associated with the overexpression of Her2/neu oncogene, whereas the overexpression of miR-30a-3p and miR-181d is associated with well-differentiated carcinomas (grade 1) compared with poorly differentiated carcinomas (grade 3), showing the diagnostic potential of multiple miRNA species for differential staging and grading of ovarian carcinoma^72^.

Aside from the expression profiling of miRNA in different tissues, blood-based serum and plasma miRNA expression is also well documented and may be used as surrogate non-invasive biomarkers. For example, miRNA expression profiling in serum and tumor-derived exosomes from the same patients revealed that eight miRNAs (miR-21, miR-141, miR-200a, miR-200b, miR-200c, miR-203, miR-205, and miR-214) are similar, both in tissue and tumor-derived exosomes^73^. Furthermore, a recent study also showed the potential of miRNAs as stable blood-based noninvasive biomarkers using serum miRNAs from EOC patients. This study reported the differential expression of eight miRNAs between cancer patients and normal controls. Among these miRNAs, five miRs, namely, miR-21, miR-92, miR-93, miR-126, and miR-29a, are significantly overexpressed, whereas three miRs, namely, miR-155, miR-127, and miR-99b, are significantly down-regulated in serum of cancer patients than their matching controls^74^. MiRNAs 21, 92, and 93 are overexpressed in patients with normal CA125 levels, further suggesting that miRNAs can be used for early diagnosis of ovarian cancer^75^. The above study strongly suggested that expression profiling of circulatory tumor-derived miRNAs can potentially be used as surrogate biomarkers for EOC detection, as well as screening of asymptomatic populations. In addition, a recent study observed the upregulation of serum miR-221 in patients with EOC compared with healthy controls, and the expression level of serum miR-221 was found to be significantly associated with stage and grade of tumor. Serum miR-221 may function as a novel diagnostic and prognostic marker and it may also serve as a potential therapeutic target in EOC treatment. MiR-200a and miR-449b are highly overexpressed in stage I than in stage III ovarian carcinoma, so they may facilitate the detection of early stage EOC^76^ (**Table 1**). ZEB1, a miR-200c target gene, does not seem to be regulated in stage I EOC, suggesting that MiR-200c may play a different role in stage I EOC^76^. A recent study suggested that plasma miR-205 and let-7f are strong biomarkers for the detection of ovarian cancer complementing CA-125. In addition, let-7f may also serve as a reliable predictive biomarker for ovarian cancer prognosis^77^. Moreover, the above studies collectively suggested that aberrant miRNA expression patterns in tissues, serum, and tumor-derived exosomes form patients with ovarian carcinoma and control samples have significant great therapeutic importance for EOC and strongly indicate that they can be used as diagnostic biomarkers for ovarian cancer patients (**Table 2**).

**Table 1:** Role of various up-regulated and down-regulated miRNAs in ovarian cancer diagnosis, prognosis, and chemoresistance

MiRNAs	Target gene	Function	Up/down-regulated	References
MiR-200 family	*ERRFI-1*	Inhibits the expression of TGF-β, ZEB1, and ZEB2	Down-regulated	^47^
MiR-200c	*ZEB1*, *ZEB2*, and *TUBB3*	MiR-200c restores chemosensitivity through inhibition of TUBB3	Up-regulated	^49^
MiR-21	*PTEN, PDCD4*	Promotes cell proliferation, invasion and migration by targeting PI3/Akt pathway	Up-regulated	^59^
MiR-214	*PTEN*	Induces cisplatin resistance through targeting PTEN	Up-regulated	^58^
MiR-100	*mTOR*, *PLK-1*	Increases sensitivity towards chemotherapy	Down-regulated	^60^
MiR-34a/b/c	*c-myc*, *CDK6*, *MET*, *E2f3*, *Bcl2*, *cyclinD1*	Reduces invasion/migration/proliferation	Down-regulated	^54^
MiR-376c	*ALK7*	Induces ALK7 and inhibits apoptosis		^62^
MiR-30c, miR-30d, miR30e-3p and miR-532-5p	*HER-2*/neu oncogene	Targets Her2/neu oncogene via MAPK (ERK1/2) pathways	Down-regulated	^72^
Let-7 family	*HMAG2*, *Min-41*, and *IMP-1*, *KRAS*, *HRAS*, *C-MYC*	Re-expression of embryonic mesenchymal genes and de-differentiation of cancer cells	Down-regulated	^36^^,^^43^
MiR-141 and miR-200a	*p38 α*	Modulate the oxidative stress response in ovarian cancer	Up-regulated	^55^

**Table 2:** Summary of the roles of miRNAs associated with diagnosis and prognosis, type of tissue taken, number of sample taken, and discovery platform used

MiRNAs	Up/down-regulated	Diagnosis/prognosis	Type of tissue	No. of samples	Discovery platform	References
MiR-21	Up-regulated	Diagnosis and prognosis both	Blood/serum	94 patients with primary EOC	Real-time RT-PCR assay	^78^
MiR-141, miR-200a/b/c	Up-regulated	Diagnosis	Tissue sample	84 snap-frozen normal and malignant ovarian tissues	MiRNA microarray hybridization	^30^^,^^40^
MiR-199, miR-140, miR-145, miR125b	Down-regulated	Diagnosis	Tissue sample	84 normal and malignant ovarian tissues	MiRNA microarray hybridization	^30^
MiR-484, miR-642, and MiR-217	Down-regulated	Prognosis	Tumor tissue	112 EOC samples	Quantitative RT-PCR	^79^
MiR-221	Up-regulated	Diagnosis and prognosis	Serum	96 patients with EOC	Real-time RT-PCR assay	^68^
MiR-31	Down-regulated	Chemoresistance	KFr13Tx cell lines	-	Real-time PCR	^67^
MiRs-21, 92, 93, 126 and 29a	Up-regulated	Diagnosis and prognosis	Serum	28 patients with EOC	TaqMan real-time PCR	^74^
MiRs-155, 127 and 99b	Down-regulated	Diagnosis and prognosis	Serum		TaqMan real-time PCR	^74^
MiR-182	Up-regulated	Prognosis (chemoresistance)	Tumor tissue as well as cell lines	15 ovarian tissue samples and 2 cell lines	Real-time PCR	^80^
MiR-23a, miR-27a, miR-21 and miR24-2	Up-regulated	Prognosis	Tumor tissue sample	56 ovarian cancer samples	Microarrays.	^75^
MiR-21, 141, 200a, 200b, 200c, 203, 205, and 214	Up-regulated	Diagnosis	Serum	50 ovarian cancer patients	MiRNA-microarrays	^73^
MiR-30c, 30d, 30e-3p, and 370	Up-regulated	Clinicopathologic variables	Ovarian cancer tissue block	171 Formalin fixed paraffin embedded tissue	Taqman based RT-PCR	^72^
MiR-221	Up-regulated	Prognosis	Serum	96 EOC samples	Real-time PCR	^81^
Let-7i	Down-regulated	Prognosis (chemotherapy response)	Tissue sample	72 patients and 4 ovarian cancer cell lines	MiRNA-microarrays	^34^
MiR-9	Down-regulated	Diagnostic for recurrent ovarian cancer	Tissue sample	18 fresh frozen and 22FFPE EOC samples	Real-time PCR	^82^
MiR-223	Up-regulated	Diagnostic for recurrent ovarian cancer	Tissue sample	18 fresh frozen and 22FFPE EOC samples	Real-time PCR	^82^

EOC, epithelial ovarian cancer.

## MiRNAs as potential prognosis marker of ovarian cancer

Several studies have reported that aberrant miRNA expression can be used as a prognostic marker to check the disease outcome during the treatment regimen. For example, in ovarian cancers, loss of let-7 and up-regulation of HMGA2 are significantly associated with unfavorable prognosis^29^. Thus, the ratio of HMGA2 to let-7 has been used for prognosis of ovarian cancer; higher ratio of HMGA2/let-7 exhibits poor survival (<10%) compared with a lower ratio (40%)^29^. In addition, let-7 used as a prognostic marker in ovarian cancer is down-regulated in chemotherapy-resistant patients and associated with shorter progression-free survival, so it may also serve as a biomarker to observe disease outcome and survival in ovarian cancer patients^34^. The prognostic value of miRNAs has also been evaluated by expression profiling in 55 ovarian cancer patients, and their subsequent expression levels are correlated with the disease outcome^83^. In this study, miR-200a showed significant association with cancer survival, and overexpression of miR-200a predicted favorable disease outcome among the 96 miRNAs analyzed^83^. However, another study showed that overexpression of miR-200a exhibits poor prognosis^84^. These contradictory results forced the requirement of large prospective studies to establish miR-200 family members as prognostic markers for ovarian cancer. MiR-9 and miR-223 are correlated with the recurrence of ovarian cancer and may be used as prognostic markers for disease outcome in ovarian cancer^82^. Recently, miR-30d has been observed to be up-regulated in patients with platinum-based chemosensitive response than in patients with chemo-resistant response. In this study, miR-30d was also observed to be down-regulated in recurrent ovarian cancer patients^72^. Furthermore, miRNA expression patterns were analyzed using tissues and cell lines of ovarian cancer; miR-221 was observed to be the most consistently overexpressed and the let-7 family members were the most frequently under-expressed miRNAs in ovarian cancers^85^. These finding suggested that these miRNAs may serve as potential biomarkers for disease outcome.

Another study analyzed the promoter methylation of let-7a-3 using 214 clinical samples of EOC^86^ and found that hypermethylation limits the risk of deaths in ovarian cancer patients. The methylation pattern of let-7a-3 is inversely correlated with the expression of IGF-II, and let-7a-3 overexpression in tumors is associated with poor prognosis^86^. Since then, a number of studies have shown the association between aberrant methylation and alterations in chromatin structure with the deregulated expression of miRNAs and consequent development of cancer. One study showed the differential expression patterns of miRNAs in serous ovarian cancer and control, and revealed that miR-21 is frequently up-regulated and miR-125b is frequently down-regulated miRNAs, in most cases^84^. The study also showed that miRNA expression between normal and malignant ovaries varies and can be used to differentiate the histotypes.

Overexpression of miR-23a, miR-27a, miR-21, and miR24-2 is significantly associated with poor prognosis in patients with progressive disease during first-line chemotherapy. Overexpression of miR-378 is associated with chemosensitization, whereas its under-expression results in chemoresistance among patients treated with platinum-based chemotherapy^75^^,^^78^. Programmed cell death 4 (PDCD4), an important tumor suppressor gene, influences transcription and translation of multiple genes through modulation of different signal transduction pathways. MiRNA-182, which directly targets PDCD4, is up-regulated in ovarian cancer tissues and cell lines. Furthermore, miR-182 reduces the chemosensitivity of ovarian cancer cells, which may be attributed to its anti-apoptotic activity to cisplatin and Taxol (**Table 2**)^80^.

The down-regulated expression of miRNA-22 (miR-22) in EOC is linked with overall survival and progression-free survival, so it may serve as an efficient prognostic factor for EOC patients^81^. Detection of miR-92 levels in the serum is an indicator of clinical stage of tumor, and it can be used as a new tumor biomarker in the diagnosis and assessment of prognosis of EOC^87^. MiRNA expression profiles also influence chemoresistance in serous epithelial ovarian carcinomas. Three miRNAs (miR-484, -642, and -217) are reportedly critical in predicting chemoresistance of these tumors. Among them, miR-484 is involved in modulating tumor vasculature by regulating the VEGFB and VEGFR2 pathways. Collectively, these three miRNAs can classify the response of ovarian cancer patients to chemotherapy in a large multicenter cohort, whereas miR-484 is involved in the control of tumor angiogenesis, indicating an option in treating patients suffering from serous epithelial ovarian carcinomas^79^.

## Future prospective

Incessant studies from the last two decades have shown that miRNAs are critical regulators of ovarian cancer and play diverse roles from EMT to cancer survival, in cell cycle regulation, for diagnosis and prognosis of cancer, and in therapeutic response to drugs and development of chemoresistance. Citing their diverse roles in each and every stage of cancer pathophysiology as evidenced by miRNA expression profiling, miRNAs can be used as ideal and highly sensitive biomarkers for different subtypes of ovarian cancers. Despite inherent technological limitations and constraints in designing studies to establish miRNAs as clinical molecular biomarkers, they still offer great hope for diagnosis, prognosis, and prediction of EOC because they resemble most of the characteristics of an ideal biomarker. Interestingly, the inherent stabilities and resilience of miRNAs in tumor tissues and blood samples are important for their applications as a reliable and rapid biomarker. The study of gene expression profiling and its clinical associations has been identified in small samples; hence, the translation of miRNAs into clinical biomarkers demands independent studies at larger scales. In addition, studies focusing on tumor-specific microvesicles are required to provide insights into the biological roles of circulating miRNAs. Apart from well-noted diagnostic and prognostic values, miRNAs also provide a potential treatment option for ovarian cancer by inhibiting the expression of pathologically relevant genes, which is mainly mediated through post-transcriptional gene silencing. Considering these facts and given the higher sensitivities and specificities of miRNAs compared with other currently used clinical biomarkers, they hold potential for use as substantial clinical biomarkers for early stage detection, monitoring progression, and therapeutics of ovarian cancer.

## Conclusion

Extensive studies from the last two decades have clearly suggested that miRNAs are critical regulators of gene expression and cell cycle machinery, thereby maintaining the differentiation status in ovarian carcinoma cells. Any deregulation in miRNA expression may trigger cancer development, cancer aggressiveness (e.g., low grade and high grade tumors), chemoresistance, migration, and metastasis. In addition, miRNA expression profiling in healthy and benign tumors may entirely differ from that in cancerous cells. Thus, expression patterns of miRNA population may serve as a prognostic and reliable biomarker for early detection, which may help in effective and improved management of ovarian carcinoma. Furthermore, identification of miRNAs associated with the different histotypes of ovarian cancer, resistance to drugs, cell invasion, and metastasis may help in designing better strategies for more individualized and efficient treatment regimens for ovarian cancer. Most of the currently available options to treat cancers are site-directed because of the limited current knowledge about disease progression and the fact that it is the most effective treatment strategy to execute. Given that identification of the primary site of tumor poses many clinical challenges and has the most significant role in treating cancers, miRNA expression profiles may improve the diagnosis of tumors from unknown primary origin, which would help in ensuring management plan and lead to better survival of ovarian cancer patients. In addition, tumor-specific and blood-based circulating miRNAs may improve cancer diagnosis and prognosis. Fortunately, several promising miRNAs have already been explored as non-invasive biomarkers for different tumor entities. At this juncture, we have only started to evaluate the role of miRNAs as clinical biomarkers for early stage diagnosis of ovarian cancer. However, before drawing any final conclusion and using miRNAs as more reliable and non-invasive biomarkers for the detection and treatment of ovarian cancer, future studies comprising large sample sets with well-supported validation through long-term clinical data must be conducted. Finally, before any clinical application of miRNAs, high-throughput optimization is critical to enhance detection under different stages and conditions of ovarian cancer pathogenesis, as well as for their reliable efficacy in cancer therapy.

## References

[1] Cramer DW. The epidemiology of endometrial and ovarian cancer. Hematol Oncol Clin North Am 2012;26:1-12. 22244658 10.1016/j.hoc.2011.10.009 PMC3259524

[2] Jemal A Siegel R Xu J Ward E. Cancer statistics. CA Cancer J Clin 2010;60:277-300. 20610543 10.3322/caac.20073

[3] Seidman JD Kurman RJ. Pathology of ovarian carcinoma. Hematol Oncol Clin North Am 2003;17:909-925. 12959182 10.1016/s0889-8588(03)00061-3

[4] Shih IM Kurman RJ. Ovarian tumorigenesis: a proposed model based on morphological and molecular genetic analysis. Am J Pathol 2004;164:1511-1518. 15111296 10.1016/s0002-9440(10)63708-x PMC1615664

[5] Zhang B Cai FF Zhong XY. An overview of biomarkers for the ovarian cancer diagnosis. Eur J Obstet Gynecol Reprod Biol 2011;158:119-123. 21632171 10.1016/j.ejogrb.2011.04.023

[6] Mor G Visintin I Lai Y Zhao H Schwartz P Rutherford T Serum protein markers for early detection of ovarian cancer. Proc Natl Acad Sci U S A 2005;102:7677-7682. 15890779 10.1073/pnas.0502178102 PMC1140439

[7] Nolen BM Lokshin AE. Biomarker testing for ovarian cancer: clinical utility of multiplex assays. Mol Diagn Ther 2013;17:139-146. 23552992 10.1007/s40291-013-0027-6 PMC3670781

[8] Jiang W Huang R Duan C Fu L Xi Y Yang Y Identification of five serum protein markers for detection of ovarian cancer by antibody arrays. PLoS One 2013;8:e76795. 24116163 10.1371/journal.pone.0076795 PMC3792870

[9] Bartel DP. MicroRNAs: genomics, biogenesis, mechanism, and function. Cell 2004;116:281-297. 14744438 10.1016/s0092-8674(04)00045-5

[10] Ambros V. The functions of animal microRNAs. Nature 2004;431:350-355. 15372042 10.1038/nature02871

[11] Perron MP Provost P. Protein interactions and complexes in human microRNA biogenesis and function. Front Biosci 2008;13:2537-2547. 17981733 10.2741/2865 PMC2901379

[12] Wightman B Ha I Ruvkun G. Posttranscriptional regulation of the heterochronic gene lin-14 by lin-4 mediates temporal pattern formation in C. elegans. Cell 1993;75:855-862. 8252622 10.1016/0092-8674(93)90530-4

[13] Lee RC Feinbaum RL Ambros V. The C. elegans heterochronic gene lin-4 encodes small RNAs with antisense complementarity to lin-14. Cell 1993;75:843-854. 8252621 10.1016/0092-8674(93)90529-y

[14] Rajewsky N. L(ou)sy miRNA targets? Nat Struct Mol Biol 2006;13:754-755. 16955093 10.1038/nsmb0906-754

[15] Rajewsky N. microRNA target predictions in animals. Nat Genet 2006;38:S8-S13. 16736023 10.1038/ng1798

[16] Liu W Mao SY Zhu WY. Impact of tiny miRNAs on cancers. World J Gastroenterol 2007;13:497-502. 17278213 10.3748/wjg.v13.i4.497 PMC4065969

[17] Berezikov E Guryev V van de Belt J Wienholds E Plasterk RH Cuppen E. Phylogenetic shadowing and computational identification of human microRNA genes. Cell 2005;120:21-24. 15652478 10.1016/j.cell.2004.12.031

[18] Stanczyk J Pedrioli DM Brentano F Sanchez-Pernaute O Kolling C Gay RE Altered expression of MicroRNA in synovial fibroblasts and synovial tissue in rheumatoid arthritis. Arthritis Rheum 2008;58:1001-1009. 18383392 10.1002/art.23386

[19] Griffiths-Jones S. miRBase: the microRNA sequence database. Methods Mol Biol 2006;342:129-138. 16957372 10.1385/1-59745-123-1:129

[20] Griffiths-Jones S. The microRNA registry. Nucleic Acids Res 2004;32:D109-D111. 14681370 10.1093/nar/gkh023 PMC308757

[21] Griffiths-Jones S Saini HK van Dongen S Enright AJ. miRBase: tools for microRNA genomics. Nucleic Acids Res 2008;36:D154-D158. 17991681 10.1093/nar/gkm952 PMC2238936

[22] Cullen BR. Transcription and processing of human microRNA precursors. Mol Cell 2004;16:861-865. 15610730 10.1016/j.molcel.2004.12.002

[23] Cho WC. MicroRNAs: potential biomarkers for cancer diagnosis, prognosis and targets for therapy. Int J Biochem Cell Biol 2010;42:1273-1281. 20026422 10.1016/j.biocel.2009.12.014

[24] Cho WC. Great potential of miRNAs as predictive and prognostic markers for cancer. Expert Rev Mol Diagn 2012;12:315-318. 22616694 10.1586/erm.12.21

[25] Baskerville S Bartel DP. Microarray profiling of microRNAs reveals frequent coexpression with neighboring miRNAs and host genes. RNA 2005;11:241-247. 15701730 10.1261/rna.7240905 PMC1370713

[26] Giannakakis A Sandaltzopoulos R Greshock J Liang S Huang J Hasegawa K miR-210 links hypoxia with cell cycle regulation and is deleted in human epithelial ovarian cancer. Cancer Biol Ther 2008;7:255-264. 18059191 10.4161/cbt.7.2.5297 PMC3233968

[27] Calin GA Sevignani C Dumitru CD Hyslop T Noch E Yendamuri S Human microRNA genes are frequently located at fragile sites and genomic regions involved in cancers. Proc Natl Acad Sci U S A 2004;101:2999-3004. 14973191 10.1073/pnas.0307323101 PMC365734

[28] Sevignani C Calin GA Nnadi SC Shimizu M Davuluri RV Hyslop T MicroRNA genes are frequently located near mouse cancer susceptibility loci. Proc Natl Acad Sci U S A 2007;104:8017-8022. 17470785 10.1073/pnas.0702177104 PMC1876564

[29] Shell S Park SM Radjabi AR Schickel R Kistner EO Jewell DA Let-7 expression defines two differentiation stages of cancer. Proc Natl Acad Sci U S A 2007;104:11400-11405. 17600087 10.1073/pnas.0704372104 PMC2040910

[30] Iorio MV Visone R Di Leva G Donati V Petrocca F Casalini P MicroRNA signatures in human ovarian cancer. Cancer Res 2007;67:8699-8707. 17875710 10.1158/0008-5472.CAN-07-1936

[31] Roush S Slack FJ. The let-7 family of microRNAs. Trends Cell Biol 2008;18:505-516. 18774294 10.1016/j.tcb.2008.07.007

[32] Pasquinelli AE Reinhart BJ Slack F Martindale MQ Kuroda MI Maller B Conservation of the sequence and temporal expression of let-7 heterochronic regulatory RNA. Nature 2000;408:86-89. 11081512 10.1038/35040556

[33] Takamizawa J Konishi H Yanagisawa K Tomida S Osada H Endoh H Reduced expression of the let-7 microRNAs in human lung cancers in association with shortened postoperative survival. Cancer Res 2004;64:3753-3756. 15172979 10.1158/0008-5472.CAN-04-0637

[34] Yang N Kaur S Volinia S Greshock J Lassus H Hasegawa K MicroRNA microarray identifies Let-7i as a novel biomarker and therapeutic target in human epithelial ovarian cancer. Cancer Res 2008;68:10307-10314. 19074899 10.1158/0008-5472.CAN-08-1954 PMC2762326

[35] Johnson SM Grosshans H Shingara J Byrom M Jarvis R Cheng A RAS is regulated by the let-7 microRNA family. Cell 2005;120:635-647. 15766527 10.1016/j.cell.2005.01.014

[36] Büssing I Slack FJ Grosshans H. Let-7 microRNAs in development, stem cells and cancer. Trends Mol Med 2008;14:400-409. 18674967 10.1016/j.molmed.2008.07.001

[37] Calin GA Dumitru CD Shimizu M Bichi R Zupo S Noch E Frequent deletions and down-regulation of micro- RNA genes miR15 and miR16 at 13q14 in chronic lymphocytic leukemia. Proc Natl Acad Sci USA 2002;99:15524-15529. 12434020 10.1073/pnas.242606799 PMC137750

[38] Chen PS Su JL Hung MC. Dysregulation of microRNAs in cancer. J Biomed Sci 2012;19:90. 23075324 10.1186/1423-0127-19-90 PMC3482395

[39] Calin GA Croce CM. MicroRNA signatures in human cancers. Nat Rev Cancer 2006;6:857-866. 17060945 10.1038/nrc1997

[40] Zhang W Dahlberg JE Tam W. MicroRNAs in tumorigenesis: a primer. Am J Pathol 2007;171:728-738. 17724137 10.2353/ajpath.2007.070070 PMC1959494

[41] Zhang L Volinia S Bonome T Calin GA Greshock J Yang N Genomic and epigenetic alterations deregulate microRNA expression in human EOC. Proc Natl Acad Sci U S A 2008;105:7004-7009. 18458333 10.1073/pnas.0801615105 PMC2383982

[42] Parikh A Lee C Joseph P Marchini S Baccarini A Kolev V microRNA-181a has a critical role in ovarian cancer progression through the regulation of the epithelial–mesenchymal transition. Nat Commun 2014;5:2977. 24394555 10.1038/ncomms3977 PMC3896774

[43] Wang X Cao L Wang Y Wang X Liu N You Y. Regulation of let-7 and its target oncogenes (Review). Oncol Lett 2012;3:955-960. 22783372 10.3892/ol.2012.609 PMC3389667

[44] Calura E Martini P Sales G Beltrame L Chiorino G D'Incalci M Wiring miRNAs to pathways: a topological approach to integrate miRNA and mRNA expression profiles. Nucleic Acids Res 2014;42:e96. 24803669 10.1093/nar/gku354 PMC4066781

[45] Hiss D. Optimizing molecular-targeted therapies in ovarian cancer: the renewed surge of interest in ovarian cancer biomarkers and cell signaling pathways. J Oncol 2012;2012:737981. 10.1155/2012/737981 PMC3306947 22481932

[46] Li SD Zhang JR Wang YQ Wan XP. The role of microRNAs in ovarian cancer initiation and progression. J Cell Mol Med 2010;14:2240-2249. 20345848 10.1111/j.1582-4934.2010.01058.x PMC3822563

[47] Gregory PA Bracken CP Bert AG Goodall GJ. MicroRNAs as regulators of epithelial-mesenchymal transition. Cell Cycle 2008;7:3112-3118. 18927505 10.4161/cc.7.20.6851

[48] Gregory PA Bert AG Paterson EL Barry SC Tsykin A Farshid G The miR-200 family and miR-205 regulate epithelial to mesenchymal transition by targeting ZEB1 and SIP1. Nat Cell Biol 2008;10:593-601. 18376396 10.1038/ncb1722

[49] Park SM Gaur AB Lengyel E Peter ME. The miR-200 family determines the epithelial phenotype of cancer cells by targeting the E-cadherin repressors ZEB1 and ZEB2. Genes Dev 2008;22:894-907. 18381893 10.1101/gad.1640608 PMC2279201

[50] Korpal M Lee ES Hu G Kang Y. The miR-200 family inhibits epithelialmesenchymal transition and cancer cell migration by direct targeting of E-cadherin transcriptional repressors ZEB1 and ZEB2. J Biol Chem 2008;283:14910-14914. 18411277 10.1074/jbc.C800074200 PMC3258899

[51] Leskelä S Leandro-García LJ Mendiola M Barriuso J Inglada-Pérez L Muñoz I The miR-200 family controls beta-tubulin III expression and is associated with paclitaxel-based treatment response and progression-free survival in ovarian cancer patients. Endocr Relat Cancer 2011;18:85-95. 21051560 10.1677/ERC-10-0148

[52] Sun N Zhang Q Xu C Zhao Q Ma Y Lu X Molecular regulation of ovarian cancer cell invasion. Tumour Biol 2014;35:11359-11366. 25119590 10.1007/s13277-014-2434-7

[53] Kim NH Kim HS Li XY Lee I Choi HS Kang SE A p53/miRNA-34 axis regulates Snail1-dependent cancer cell epithelial–mesenchymal transition. J Cell Biol 2011;195:417-433. 22024162 10.1083/jcb.201103097 PMC3206336

[54] Corney DC Hwang CI Matoso A Vogt M Flesken-Nikitin A Godwin AK Frequent downregulation of miR-34 family in human ovarian cancers. Clin Cancer Res 2010;16:1119-1128. 20145172 10.1158/1078-0432.CCR-09-2642 PMC2822884

[55] Mateescu B Batista L Cardon M Gruosso T de Feraudy Y Mariani O miR-141 and miR-200a act on ovarian tumorigenesis by controlling oxidative stress response. Nat Med 2011;17:1627-1635. 22101765 10.1038/nm.2512

[56] Park SM Shell S Radjabi AR Schickel R Feig C Boyerinas B Let-7 prevents early cancer progression by suppressing expression of the embryonic gene HMGA2. Cell Cycle 2007;6:2585-2590. 17957144 10.4161/cc.6.21.4845

[57] Johnson CD Esquela-Kerscher A Stefani G Byrom M Kelnar K Ovcharenko D The let-7 microRNA represses cell proliferation pathways in human cells. Cancer Res 2007;67:7713-7722. 17699775 10.1158/0008-5472.CAN-07-1083

[58] Yang H Kong W He L Zhao JJ O'Donnell JD Wang J MicroRNA expression profiling in human ovarian cancer: miR-214 induces cell survival and cisplatin resistance by targeting PTEN. Cancer Res 2008;68:425-433. 18199536 10.1158/0008-5472.CAN-07-2488

[59] Lou Y Yang X Wang F Cui Z Huang Y. MicroRNA-21 promotes the cell proliferation, invasion and migration abilities in ovarian epithelial carcinomas through inhibiting the expression of PTEN protein. Int J Mol Med 2010;26:819-827. 21042775 10.3892/ijmm_00000530

[60] Nagaraja AK Creighton CJ Yu Z Zhu H Gunaratne PH Reid JG. A link between mir-100 and FRAP1/mTOR in clear cell ovarian cancer. Mol Endocrinol 2010;24:447-463. 20081105 10.1210/me.2009-0295 PMC2817607

[61] Peng DX Luo M Qiu LW He YL Wang XF. Prognostic implications of microRNA-100 and its functional roles in human epithelial ovarian cancer. Oncol Rep 2012;27:1238-1244. 22246341 10.3892/or.2012.1625 PMC3583406

[62] Ye G Fu G Cui S Zhao S Bernaudo S Bai Y MicroRNA 376c enhances ovarian cancer cell survival by targeting activin receptor-like kinase 7: implications for chemoresistance. J Cell Sci 2011;124:359-368. 21224400 10.1242/jcs.072223

[63] Fang L Deng Z Shatseva T Yang J Peng C Du WW MicroRNA miR-93 promotes tumor growth and angiogenesis by targeting integrin-beta8. Oncogene 2011;30:806-821. 20956944 10.1038/onc.2010.465

[64] Nakano H Yamada Y Miyazawa T Yoshida T. Gain-of-function microRNA screens identify miR-193a regulating proliferation and apoptosis in epithelial ovarian cancer cells. Int J Oncol 2013;42:1875-1882. 23588298 10.3892/ijo.2013.1896 PMC3699598

[65] Liu L Zou J Wang Q Yin FQ Zhang W Li L. Novel microRNAs expression of patients with chemotherapy drug-resistant and chemotherapy-sensitive epithelial ovarian cancer. Tumour Biol 2014;35:7713-7717. 24805828 10.1007/s13277-014-1970-5

[66] Yang D Sun Y Hu L Zheng H Ji P Pecot CV Integrated analyses identify a master microrna regulatory network for the mesenchymal subtype in serous ovarian cancer. Cancer Cell 2013;23:186-199. 23410973 10.1016/j.ccr.2012.12.020 PMC3603369

[67] Creighton CJ Fountain MD Yu Z Nagaraja AK Zhu H Khan M Molecular profiling uncovers a p53-associated role for microRNA-31 in inhibiting the proliferation of serous ovarian carcinomas and other cancers. Cancer Res 2010;70:1906-1915. 20179198 10.1158/0008-5472.CAN-09-3875 PMC2831102

[68] Medina R Zaidi SK Liu CG Stein JL van Wijnen AJ Croce CM MicroRNAs 221 and 222 bypass quiescence and compromise cell survival. Cancer Res 2008;68:2773-2780. 18413744 10.1158/0008-5472.CAN-07-6754 PMC3613850

[69] Li Y Yao L Liu F Hong J Chen L Zhang B Characterization of microRNA expression in serous ovarian carcinoma. Int J Mol Med 2014;34:491-498. 24939816 10.3892/ijmm.2014.1813

[70] Liang H Jiang Z Xie G Lu Y. Serum microRNA-145 as a novel biomarker in human ovarian cancer. Tumour Biol 2015;36:5305-5313. 25722112 10.1007/s13277-015-3191-y

[71] Wyman SK Parkin RK Mitchell PS Fritz BR O'Briant K Godwin AK Repertoire of microRNAs in epithelial ovarian cancer as determined by next generation sequencing of small RNA cDNA libraries. PLoS One 2009;4:e5311. 19390579 10.1371/journal.pone.0005311 PMC2668797

[72] Lee H Park CS Deftereos G Morihara J Stern JE Hawes SE MicroRNA expression in ovarian carcinoma and its correlation with clinicopathological features. World J Surg Oncol 2012;10:174. 22925189 10.1186/1477-7819-10-174 PMC3449188

[73] Taylor DD Gercel-Taylor C. MicroRNA signatures of tumor-derived exosomes as diagnostic biomarkers of ovarian cancer. Gynecol Oncol 2008;110:13-21. 18589210 10.1016/j.ygyno.2008.04.033

[74] Resnick KE Alder H Hagan JP Richardson DL Croce CM Cohn DE. The detection of differentially expressed microRNAs from the serum of ovarian cancer patients using a novel real-time PCR platform. Gynecol Oncol 2009;112:55-59. 18954897 10.1016/j.ygyno.2008.08.036

[75] Eitan R Kushnir M Lithwick-Yanai G David MB Hoshen M Glezerman M Tumor microRNA expression patterns associated with resistance to platinum based chemotherapy and survival in ovarian cancer patients. Gynecol Oncol 2009;114:253-259. 19446316 10.1016/j.ygyno.2009.04.024

[76] Marchini S Cavalieri D Fruscio R Calura E Garavaglia D Fuso Nerini I Association between miR-200c and the survival of patients with stage I epithelial ovarian cancer: a retrospective study of two independent tumour tissue collections. Lancet Oncol 2011;12:273-285. 21345725 10.1016/S1470-2045(11)70012-2

[77] Zheng H Zhang L Zhao Y Yang D Song F Wen Y Plasma miRNAs as diagnostic and prognostic biomarkers for ovarian cancer. PLoS One 2013;8:e77853. 24223734 10.1371/journal.pone.0077853 PMC3815222

[78] Xu YZ Xi QH Ge WL Zhang XQ. Identification of serum microRNA-21 as a biomarker for early detection and prognosis in human epithelial ovarian cancer. Asian Pac J Cancer Prev 2013;14:1057-1060. 23621186 10.7314/apjcp.2013.14.2.1057

[79] Vecchione A Bellettic B Lovatb F Volinia S Chiappetta G Giglio S A microRNA signature defines chemoresistance in ovarian cancer through modulation of angiogenesis. Proc Natl Acad Sci U S A 2013;110:9845-9850. 23697367 10.1073/pnas.1305472110 PMC3683704

[80] Wang YQ Guo RD Guo RM Sheng W Yin LR. MicroRNA-182 promotes cell growth, invasion and chemoresistance by targeting programmed cell death 4 (PDCD4) in human ovarian carcinomas. J Cell Biochem 2013;114:1464-1473. 23296900 10.1002/jcb.24488

[81] Wan WN Zhang YQ Wang XM Liu YJ Zhang YX Que YH Down-regulated miR-22 as predictive biomarkers for prognosis of epithelial ovarian cancer. Diagnostic Pathology 2014;9:178. 25257702 10.1186/s13000-014-0178-8 PMC4180346

[82] Laios A O’Toole S Flavin R Martin C Kelly L Ring M Potential role of miR-9 and miR-223 in recurrent ovarian cancer. Mol Cancer 2008;7:35. 18442408 10.1186/1476-4598-7-35 PMC2383925

[83] Hu X Macdonald DM Huettner PC Feng Z El Naqa IM Schwarz JK A miR-200 microRNA cluster as prognostic marker in advanced ovarian cancer. Gynecol Oncol 2009;114:457-464. 19501389 10.1016/j.ygyno.2009.05.022

[84] Nam EJ Yoon H Kim SW Kim H Kim YT Kim JH MicroRNAs expression profiles in serous ovarian carcinoma. Clin Cancer Res 2008;14:2690-2695. 18451233 10.1158/1078-0432.CCR-07-1731

[85] Dahiya N Sherman-Baust CA Wang TL Davidson B Shih IeM Zhang Y MicroRNA expression and identification of putative miRNA targets in ovarian cancer. PLoS One 2008;3:e2436. 18560586 10.1371/journal.pone.0002436 PMC2410296

[86] Lu L Katsaros D de la Longrais IA Sochirca O Yu H. Hypermethylation of let-7a-3 in epithelial ovarian cancer is associated with low insulin-like growth factor-II expression and favorable prognosis. Cancer Res 2007;67:10117-10122. 17974952 10.1158/0008-5472.CAN-07-2544

[87] Guo F Tian J Lin Y Jin Y Wang L Cui M Serum microRNA-92 expression in patients with ovarian epithelial carcinoma. J Int Med Res 2013;41:1456-1461. 23963852 10.1177/0300060513487652

